# Chemical and Microbial Characterization of Fermented Forest Litters Used as Biofertilizers

**DOI:** 10.3390/microorganisms11020306

**Published:** 2023-01-24

**Authors:** Johann Marois, Thomas Z. Lerch, Ugo Dunant, Anne-Marie Farnet Da Silva, Pierre Christen

**Affiliations:** 1 Institute of Ecology and Environnemental Sciences of Paris, UMR 7618 (CNRS, SU, IRD, UPEC, INRAe, UPC), 94010 Créteil, France; 2 Institut Méditerranéen de Biodiversité et d’Ecologie marine et Continentale, UMR 7263 (CNRS, AMU, IRD, AU), 13397 Marseille, France

**Keywords:** biofertilizers, fermented forest litters, microbial communities, organic matter composition

## Abstract

The excessive use of chemicals in intensive agriculture has had a negative impact on soil diversity and fertility. A strategy for developing sustainable agriculture could rely on the use of microbial-based fertilizers, known as biofertilizers. An alternative to marketed products could be offered to small farmers if they could produce their own biofertilizers using forest litters, which harbor one of the highest microbial diversities. The aim of this study is to characterize microbial communities of Fermented Forest Litters (FFL), assuming that the fermentation process will change both their abundance and diversity. We investigated two types of differing in the chemical composition of the initial litters used and the climatic context of the forest where they are originated from. The abundance and diversity of bacterial and fungal communities were assessed using quantitative PCR and molecular genotyping techniques. The litter chemical compositions were compared before and after fermentation using Infrared spectrometry. Results obtained showed that fermentation increased the abundance of bacteria but decreased that of fungi. Low pH and change in organic matter composition observed after fermentation also significantly reduced the α-diversity of both bacterial and fungal communities. The higher proportion of aliphatic molecules and lower C/N of the FFLs compared to initial litters indicate that FFLs should be rapidly decomposed once added into the soil. This preliminary study suggests that the agronomic interest of FFLs used as biofertilizers is probably more related to the contribution of nutrients easily assimilated by plants than to the diversity of microorganisms that compose it. Further studies must be conducted with sequencing techniques to identify precisely the microbial species likely to be beneficial to plant growth.

## 1. Introduction

The “Green Revolution”, which began just before and after the Second World War, led to a tremendous increase in crop yields worldwide. This was due to a combination of three factors: the use of ‘selected’ seeds of high-yielding varieties, the use of fertilizers and plant protection products and a favorable policy environment for market regulation. For more than half a century, increased pressure on food production has led to a constant quest to improve agricultural yields, resulting in the ever-increasing use of chemical fertilizers, pesticides and other agrochemicals. Excessive use of these chemicals has many negative effects on agroecosystems, including destruction of soil structure and biological activity, acidification, reduced nutrient use efficiency and altered biodiversity [1,2,3]. For several years, this global awareness has been calling for new alternatives to move towards a “Green Revolution 2.0” [4]. Several strategies have been proposed and evaluated over the years to both recycle organic waste of all types and improve soil health e.g., [5,6,7]. Another strategy for developing sustainable agriculture could be to use microbial-based fertilizers, otherwise known as biofertilizers [8].

The term biofertilizer refers to a formulation comprising a single strain or microbial community that allows for increased plant growth through improved nutrient availability [9]. Most often, but not always, these formulations are based on the use of plant growth-promoting bacteria or rhizobacteria (PGPB/PGPR) which colonize roots after inoculation and enhance the nutrition of host plants [10]. Biofertilizers can also provide other direct and indirect benefits for plant growth, such as phytostimulation, abiotic stress tolerance and biocontrol [11,12,13]. The discovery of the beneficial effects of microorganisms on plants is not recent, as in 1895, Nobbe and Hiltner already used Rhizobium sp. as biofertilizers [14]. However, the understanding of the links between microbial diversity and functioning in soils, and their relationships with plants, has really progressed thanks to technological advances in recent years [15]. Thus, through the enormous potential that microorganisms can offer in agroecology, bioinoculants can contribute to achieving the goals of sustainable agriculture [16].

Although the biofertilizer market is increasing [17,18], the cost of production and utilization may still be too high for small farmers in developed countries, just like conventional fertilizers. An alternative to marketed products could be offered to them if they could produce their own biofertilizers. Such a process has been developed and tested in the Experimental Station Indio Hatuey (ESIH) from the Matanzas University of Cuba (unpublished data). This process consists of two successive fermentation steps to produce liquid containing beneficial microorganisms originated from forest litter. Indeed, the forest microbiome is one of the most diverse, both in terms of bacterial and fungal communities [19]. The first fermentation takes place in the solid phase and use of forest litter with whey as the bacterial source for the anaerobic fermentation [20], a source of sugar, a source of cellulosic compound and water. After one month, the fermentation product serves to inoculate a liquid state for the second fermentation, also called activation. This process has the advantage to be easy, low cost and created from native microorganisms from local litter forest. These native microorganisms are supposed to be more adapted to local conditions than commercialized bioinoculants [21]. Despite several farmers already using this biofertilizer known as “fermented forest litter” (FFL), there is still a lack of knowledge about the chemical and microbial characterization of this product. In this study, we investigated two types of FFL differing in the initial litters used and climatic context. Indeed, it has been shown that mixing litters from different trees can potentially maximize the diversity of nutrient resources and consequently, favor microbial diversity [22], but this effect strongly varied according to the climate context [23,24]. Here, we hypothesized that the more chemically dissimilar the litters used, the higher the microbial diversity in the FFL. To test this hypothesis, the chemical composition of litters was investigated using elementary analysis and mid-infrared spectrometry; bacterial and fungal communities were quantified by qPCR and characterized using genotyping in the initial litter mixtures before and after fermentation.

## 2. Materials and Methods

### 2.1. Forest Litter Sampling and Fermentation Process

Two forest litters’ mixtures from different climates were studied. The first mixture of litters (Mix1) was made with 37% of white oak (WO) and 63% of holm oak and aleppo pine (OP) collected in forests under a Mediterranean climate (Marseille and Sainte-Baume, France). The second mixture of litters was made with 22% of chestnut three (CN), 22% of holmù oak (HO), 26% of maritime pine (MP), 14% of hackberry tree (HB) and 16% of bamboo (BB) collected in forests under a more temperate climate (Ardeche, France). FFL were produced as recommended by the GNO Terre & Humanisme [25]. Before the fermentation, the two mixtures (Mix1 and Mix2) were obtained by adding 7.4 kg of each forest litters mix to 9.3 kg of wheat bran, 2.6 kg of sugarcane molasse, 2.8 kg of whey and 2.3 L of distilled water (Figure S1). After mixing all components, the mixtures were placed into a 32 L (Ø39 cm × 32 cm) canister closed hermetically and left to ferment in an anaerobic condition for 4 weeks (FMix1 and FMix2) at room temperature (varying from 15 °C to 25 °C). This period was determined by following the evolution of the drop in pH, which stabilizes after 3 weeks. The final product is usually diluted with water to 5% before application into the fields. The chemical and microbial measurements described below were performed on samples (100 g) collected on each replicate (n = 3) of the initial litters’ mixtures before and after fermentation.

### 2.2. Physico-Chemical Analyses

The pH_Water_ and electric conductivity of the different litters was determined on a 1:5 soil:deionized water suspension using a laboratory bench meter (HI5221, Hanna Instruments). After sieving at <200 μm, litters OC and N contents were measured using an Elemental Analyzer (Carlo Erba NA 1500). A subsample was used to characterize the organic matter by mid infrared spectroscopy (MIR) using a Fourier Transform Spectrophotometer (FTIR 660, Agilent Technologies, Santa Clara, CA, USA), operating in diffuse reflectance mode. Measurements were made at 2 cm^−1^ intervals and converted to absorbance. Only spectra from 3700 to 424 cm^−1^ were selected for data analysis in order to disregard the noise at the two edges of the spectral range. We also removed bands associated with CO_2_ interference (2500–2300 cm^−1^). We centered all means of the spectra before the multivariate analyses. The humification index (HI) [26] was computed as the ratio of aliphatic absorbance intensity at 3010–2810 cm^−^^1^ and the absorbance intensity of aromatic C=C, ketone and quinone C=O, and/or amide C=O at 1660–1580 cm^−^^1^

### 2.3. DNA Extraction and Quantification

The DNA extraction was performed on 125 mg of litter with the NucleoSpin Soil kit by Macherey-Nagel (Düren, Germany) following the instructions of the manufacturer. The lysing buffers SL2 and SX were chosen. The DNA quantification was carried out using the Broad-Range Quant-iT™ kit (Molecular Probes, Invitrogen, Paisley, UK) using a 96-well plate. The fluorescence was measured with a microplate reader Synergy™ HTX (BioTek Instruments, Winooski, VT, USA). In addition, the purity of the DNA was estimated by measuring the 260/280 nm and 260/230 nm ratios using a NanoDrop 1000 spectrophotometer (Thermo Scientific, Wilmington, DE, USA). To avoid differential efficiency of the Taq polymerase, all DNA extracts were then diluted to 0.5 ng μL^−^^1^ for subsequent analyses.

### 2.4. Quantification of Microbial Communities by qPCR

Quantification of soil microbial communities was performed by real-time PCR amplification targeting the 16S rRNA gene for bacteria and internal transcribed spacer (ITS) gene for fungi as described in Nunan et al. (2015) [27]. Briefly, each reaction was carried out using previously described primers 341F/534R [28] for 16S rRNA and ITS3/ITS4 [29] for ITS. qPCR was performed on a CFX Connect thermocycler (BioRad) following the following conditions: initial denaturation step at 94 °C for 4 min, followed by 40 cycles of denaturation at 95 °C for 10 s, annealing at 60 °C (16S rRNA) or 55 °C (ITS) for 30 s, extension at 72 °C for 10 s. Results were analyzed using CFX Manager™ software. The abundance of each gene of interest was expressed in copy numbers per gram of litter taking into account the dilution factor of the DNA extracts and soil moisture. As several PCR inhibitors may be responsible for artefactual but significant reduction of genes’ copy number quantification, an inhibition test was performed by spiking DNA extracts from all litters with control plasmid DNA. Briefly, 2 µL of cloning vector TA pCR™4-TOPO^®^ (ThermoFischer) at 10^−3^ ng µL^−1^ were mixed with 7.5 µL of Power SYBR^®^ Green PCR Master Mix, 1.5 µL of M13 forward and reverse primers and 2 µL of each sample DNA extract in a total volume of 15 µL. The signal fluorescence was the same for the control plasmid DNA, both in the presence and in the absence of DNA extracted from the litter samples, indicating the absence of detectable inhibition.

### 2.5. Molecular Profiling of Microbial Communities

Bacterial and fungal community structures were analyzed using T-RFLP and ARISA fingerprinting as described by Moni et al. (2015) [30]. Briefly, bacterial 16S rRNA genes were amplified using primers FAM labelled 63F and 1389R and fungal ITS genes using primers Yakima Yellow^®^ labelled ITS1F and ITS4. A BioRad T100 thermal cycler was used for the amplification with the following programs for T-RFLP: initial denaturation at 94 °C for 2 min, followed by 30 cycles of 94 °C for 30 s, 57 °C for 45 s and 72 °C for 90 s, followed by a final extension time at 72 °C for 10 min. For ARISA, PCR conditions consisted of an initial denaturation at 95 °C for 5 min, followed by 35 cycles of 95 °C for 30 s, 55 °C for 30 s and 72 °C for 60 s, followed by a final extension time at 72 °C for 10 min. Bacterial PCR products were digested with restriction enzyme AluI (Thermo Fisher). After a desalting step, 2 µL of PCR products were mixed with formamide containing 0.5% of LIZ500 (T-RFLP) or LIZ1500 (ARISA) internal size standard (Applied Biosystems,) and denatured at 94 °C for 3 min. Samples were electrophoresed on an ABI 3730 PRISM^®^ capillary DNA sequencer (Applied Biosystems). Genetic profiles were analyzed using GeneMapper^®^ v3.7 software (Applied Biosystems) as described by Blaud et al. (2015) [31]. The richness of the genetic profiles was expressed as the total number of different restriction fragments (T-RFLP) or amplicons (ARISA), and the evenness of profiles was estimated using the Simpson-Yule index: E = 1/∑pi^2^, where pi is the proportion of a given peak. The β-diversity was estimated by using the relative abundance matrices and multivariate analyses (see below). These two parameters were first used to compare the genetic profiles among treatments but not used as exact indicators of the soil bacterial community α-diversity. The β-diversity was estimated by using the relative abundance matrices and multivariate analyses (see below).

### 2.6. Statistical Analysis

All statistical analyses were performed with R software (version 2.12.0 R Development Core Team. 2008). After normality verification (Shapiro test), comparison among treatments was performed with the analysis of variance (ANOVA) followed by the Tuckey HSD test using the “agricolae” package. Chemical (FTIR) and genetic (TRFLP/ARISA) profiling were analyzed using Principal Component Analysis followed by Between-Class Analysis (BCA) using the “ade4TkGUI” package in order to identify factors most responsible for the observed clustering in the microbial communities. The statistical validity of the association between treatment and variance of profiles was tested for significant relationships with the Monte Carlo Permutation Test with 999 permutations. In addition, the similarity of distances matrices obtained with chemical (FTIR) and genetic (TRFLP/ARISA) profiling was determined using a Mantel test performed on 1000 permutations using the “vegan” package. All graphic representations were performed using SigmaPlot 14.0 software.

## 3. Results and Discussion

### 3.1. Physico-Chemical Characteristics

The physico-chemical data obtained on forest litter and mixtures before or after fermentation differ significantly (Table 1). Most of the forest litters have slightly acidic pH values, even acidic as for maritime pine (MP), except for white oak (WO). The initial mixing before and after fermentation causes a significant increase in acidity, similarly in both mixtures. These lower pH values could be attributed to the addition of whey into the litter mix and the subsequent fermentation process. Electrical conductivity was higher in the first litter mix than in the second one, probably due to the salinity in the Mediterranean climate that can be 3 times higher in the litter compared to inland context [30]. The initial mixing before and after fermentation causes a significant increase in electrical conductivity, especially for the second mixture. This could be explained by the addition of ions contained in the co-products.

The C and N contents of the forest litter varied slightly, except for the maritime pine litter, whose C/N ratio was twice as high, as previously reported [32]. The mixture with bran, whey and molasses significantly decreases the C/N ratio with the addition of compounds with high N content. After fermentation, the two mixtures are very similar in terms of C and N contents. The lower values of the C/N ratio in the FFL compared to the initial forest litters suggest a higher N mineralization once added into the soil [31]. The humification index (HI) increases significantly with the mixture of litter and the added co-products but does not change after fermentation. This change in HI indicates that there is a higher proportion of aliphatic molecules in the FFLs compared to the original litters, which also suggests that these biofertilizers should be rapidly decomposed and thus provide available nutrients for plants.

### 3.2. Microbial Abundances

**Figure 1:**
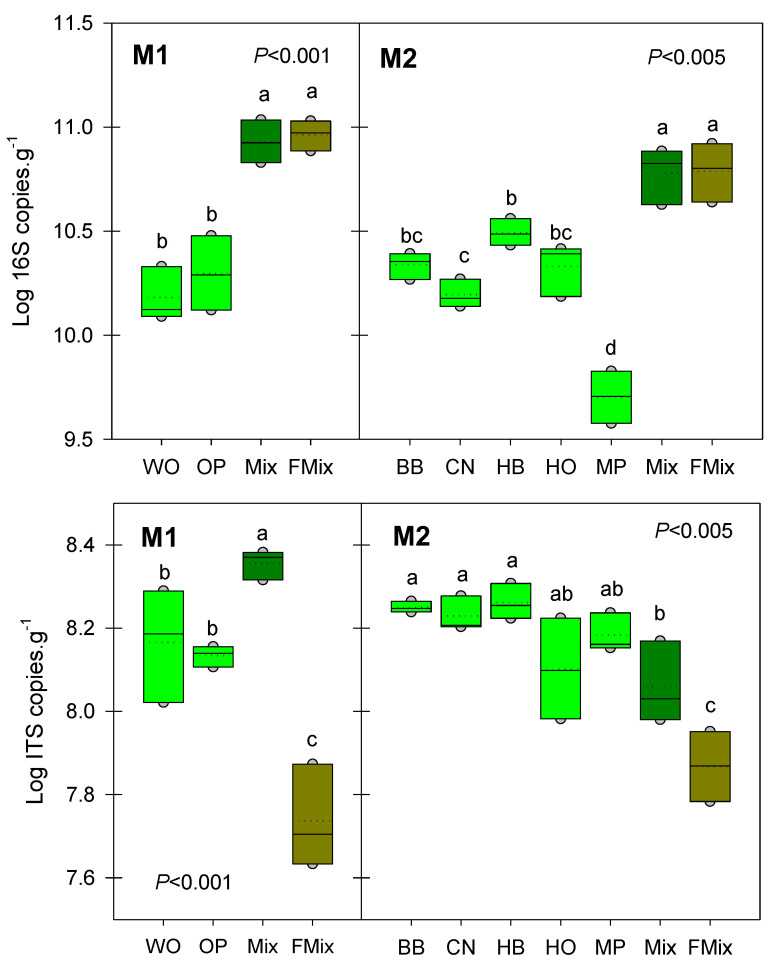
Differences in bacterial (**above**) or fungal (**below**) abundance in the two mixtures of forest litters M1 (WO: white oak, OP: holm oak and Aleppo pine) and M2 (BB: bamboo, CN: chestnut, HB: hackberry, HO: holm oak, MP: maritime pine). Mix: mixture of litters before fermentation, FMix: mixture of litters after fermentation. Different letters above the boxes indicate significant differences (*p* < 0.001) among litters.

The abundance of microbial communities evolves in a similar way during the FFL production process in the two mixtures studied (Figure 1). For the first mixture, the initial litter from Mediterranean forests shows similar abundances of bacteria and fungi. In the second mixture, the initial litter from temperate forests showed more variability, with ten times more bacteria in the hackberry tree (HB) litter than in the maritime pine (MP) litter. In both mixtures, the addition of bran, whey and molasses significantly increased the abundance of bacteria to about 10^11^ copies of 16S g^−^^1^ litter. Concerning fungi, the initial mixture tends to increase their abundance in the first mixture but slightly decreases it in the second. In both cases, fermentation considerably decreases their abundance to below 10^8^ ITS copies of g^−^^1^ litter. As a consequence, the bacteria to fungi ratio increases dramatically in the mixture after fermentation from two to three order of magnitude (Figure S2). Fermentation, therefore, has a beneficial effect on bacterial abundance but a limiting effect on a fungal one. The factor that best explains this trend is the C/N ratio. Indeed, a negative and significant correlation (Figure S3) was found between the bacteria to fungi ratio and the carbon to nitrogen ratio. It is well known that the high C/N ratio favored fungal presence, while low C/N favored dominance of bacterial populations [33]. The less initial C/N ratio of a substrate, the higher the fraction of organic N mineralized [34,35]. Therefore, the low C/N ratio of FFLs should enhance the degradation of the biofertilizers and easily release nutrients to the plants.

### 3.3. Microbial α-Diversities

**Figure 2:**
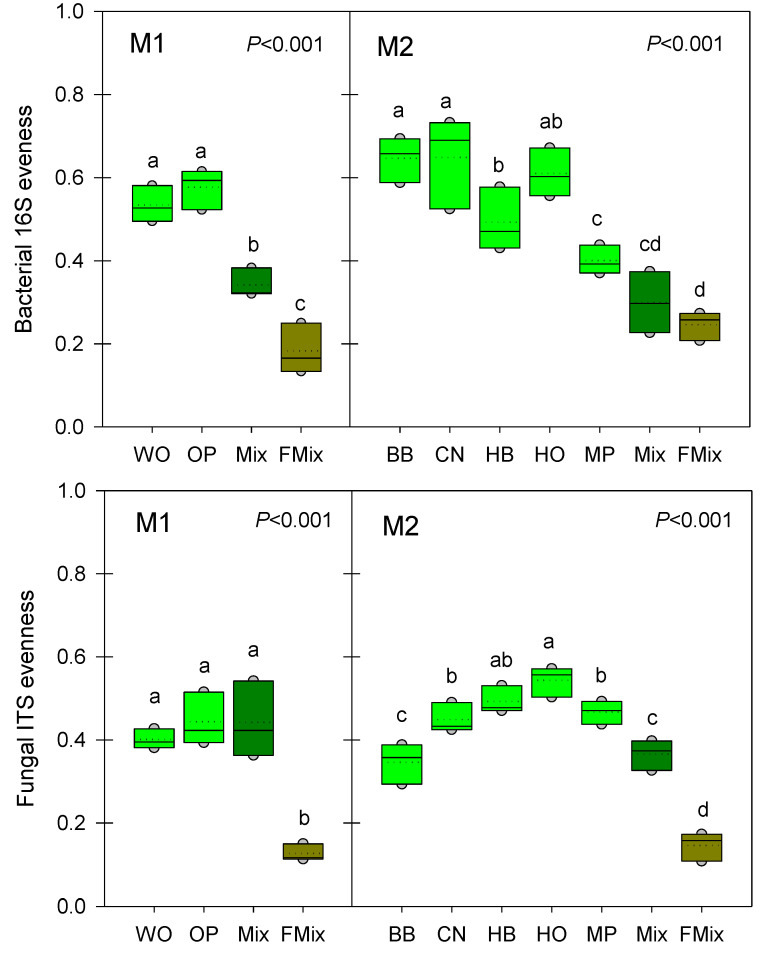
Differences in bacterial (**above**) or fungal (**below**) diversity in the two mixtures of forest litters M1 (WO: white oak, OP: holm oak and Aleppo pine) and M2 (BB: bamboo, CN: chestnut, HB: hackberry, HO: holm oak, MP: maritime pine). Mix: mixture of litters before fermentation, FMix: mixture of litters after fermentation. Different letters above the boxes indicate significant differences (*p* < 0.001) among litters.

As for the abundance, the indicators of α-diversity indicate that the microbial communities are strongly impacted during the FFL production process, with similitudes between the two mixtures studied (Figure 2 and Figure S4). For the first mixture, the number of bacterial TRF (Terminal-Restriction Fragment) found in the initial litter from Mediterranean forests were between 33 and 38 in average, more than 40 after mixing with bran, whey and molasses, and finally drop to 25 after fermentation (Figure S2). Although a higher variability was found in TRF richness, the same trend was observed in the second mixture preparation. The bacterial community evenness indexes evolved similarly in both mixtures, with a decrease from around 0.6 to 0.2 after the fermentation process, indicating a strong selection of bacterial species. Concerning fungi, this selection was even stronger than for bacteria as indicated by the high decrease in both ITS amplicons richness and evenness. This selection is probably attributed to the decrease of the pH and changes in organic matter composition. These results are consistent with other studies, although performed on different plant materials. For example, the richness and effective number of bacterial and fungal genera during medicinal plants tend to decrease during fermentation [36]. Further investigation using high throughput sequencing could be performed in order to identify the most dominant species in the FFLs. Due to the fermentation process, we expect a high abundance of Lactic acid bacteria (LAB), that are known to promote plant growth and are often found in commercial biofertilizers [37].

### 3.4. Microbial β-Diversities

**Figure 3:**
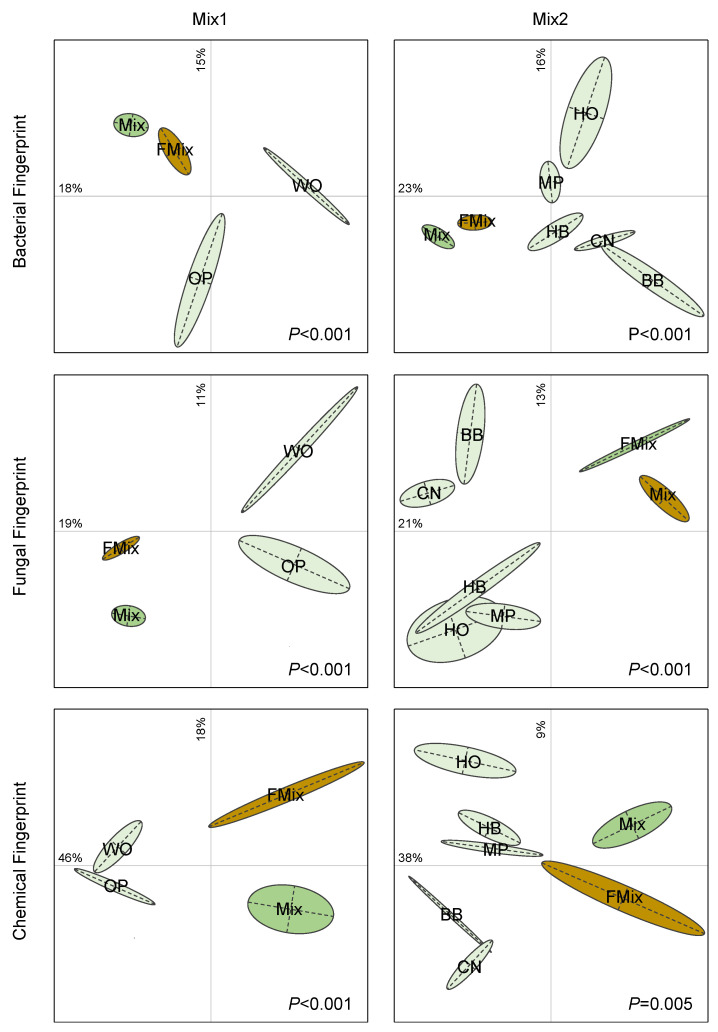
Differences in bacterial, fungal and chemical composition of the two mixtures of forest litters. Mix1 (WO: white oak, OP: holm oak and Aleppo pine) and Mix2 (BB: bamboo, CN: chestnut, HB: hackberry, HO: holm oak, MP: maritime pine). Mix: mixture of litters before fermentation, FMix: mixture of litters after fermentation). Between Class Analysis (BCA) is based on bacterial (**above**) or fungal (**middle**) molecular fingerprints or MIR spectra (**below**). The ellipses represent 60% of the variability of a litter. Letters represent the barycenter of the replicates (n = 3) for each treatment. Monte Carlo test simulated *p* values (lower right corner) revealed significant differences among litters.

The multivariate analyses performed on TRFLP and ARISA profiles showed significant differences among the litters, both for bacterial and fungal communities (Figure 3). In the first mix, the initial forest litters were clearly separated from the FFL on the first two components of the BCA, which explained 33% of the variability. The same trend was also found for the second mixture with 39% of the variability explained on the first two components of the BCA. Concerning fungi, the BCA explained 30 and 34% of the variability, for the first and second mixtures, respectively. These results suggest that both fungal and bacterial communities’ compositions are clearly different from the initial communities found in the litters collected in the forests. Besides, the multivariate analyses performed on MIR spectra (Figure 3) showed that the chemical composition of litters, mixtures before and after fermentation, are very different. Changes in microbial structures can be attributed to the chemical modification during fermentation, as suggested to the significant relationships found between molecular fingerprints and the infrared spectra (r*^2^* = 0.61 and r*^2^* = 0.70, respectively, for bacterial and fungal communities). A similar microbial and chemical composition was found between the two FFL (Figure S5), suggesting that the initial diversity of litters do not influence the final products.

## 4. Conclusions

Fermented Forest Litters (FFL) are biofertilizers which could represent an alternative to marketed products for small farmers. However, there is no chemical or microbial characterization of these products so far. In this study, we showed that the fermentation process changed the chemical composition as well as the microbial community structures of the initial forest litters. Contrary to our hypothesis, the initial differences among the litters, whether chemical or microbiological, are mitigated by the fermentation process. Here, we did not observe any differences between the two FFLs, whatever the origin of the litters or the complexity of the mixture. Due to their low C/N ratio and high proportion of aliphatic molecules, FFLs should be rapidly decomposed in soils and release nutrients available for plants. However, the fermentation process causes a drastic loss of diversity, which potentially leads to a select few beneficial microorganisms. Further studies should be conducted in order to know if the effect of FFL on soil fertility is more due to the nutrients present in the mixture and/or to the presence of potential beneficial microorganisms. Beyond the identification of these microorganisms using a sequencing method, an important point is to know if they can survive in an aerobic and oligotrophic habitat, such as soil, since they have been selected by anaerobic and copriotrophic conditions.

## Figures and Tables

**Table 1:** Physico-chemical and chemical properties of the two mixtures (M1 and M2) of forest litters (WO: white oak, OP: holm oak and alepo pine, BB: bamboo, CN: chestnut, HB: hackberry, HO: holm oak, MP: maritime pine, Mix: mixture of litters before fermentation, FMix: mixture of litters after fermentation). Values are the mean of three replicates. Ec is the electric conductivity and HI the humification index. Different lowercase letters indicate significant (*p* < 0.01) differences among treatments.

		pH_Water_	Ec (μS.cm^−1^)	C(%)	N(%)	C/N	HI
Mix1:	WO	7.12 ^a^	175 ^c^	50.37 ^a^	1.82 ^c^	27.58 ^a^	0.89 ^b^
	OP	6.78 ^b^	169 ^c^	52.95 ^a^	2.61 ^b^	20.77 ^b^	0.90 ^b^
	Mix1	6.31 ^c^	1238 ^b^	53.06 ^a^	2.90 ^ab^	18.24 ^bc^	0.96 ^a^
	FMix1	4.29 ^d^	2134 ^a^	52.36 ^a^	3.39 ^a^	15.43 ^c^	0.97 ^a^
Mix2:	BB	6.58 ^a^	71 ^d^	38.18 ^cd^	1.49 ^b^	25.77 ^bc^	0.89 ^d^
	CN	6.41 ^a^	88 ^cd^	54.04 ^ab^	1.54 ^b^	35.01 ^b^	0.92 ^bc^
	HB	6.61 ^a^	34 ^e^	44.85 ^bc^	1.75 ^b^	25.61 ^bc^	0.91 ^cd^
	HO	5.38 ^b^	95 ^c^	34.40 ^d^	1.58 ^b^	24.52 ^cd^	0.88 ^d^
	MP	4.62 ^c^	92 ^c^	57.12 ^a^	0.84 ^c^	68.54 ^a^	0.95 ^ab^
	Mix2	5.78 ^b^	2219 ^b^	51.67 ^ab^	3.02 ^a^	17.11 ^cd^	0.97 ^a^
	FMix2	4.36 ^d^	2876 ^a^	52.03 ^ab^	3.44 ^a^	15.10 ^d^	0.96 ^ab^

## Data Availability

The data presented in this study are available on request from the corresponding author.

## Supplementary Materials

The following supporting information can be downloaded at: https://www.mdpi.com/article/10.3390/microorganisms11020306/s1, Figure S1. Flowchart representing the different steps of the process, from the mixture of forest litters, addition of wheat bran, whey and sugarcane molasses, to the anaerobic fermentation during 4 weeks. Figure S2. Differences in bacterial to fungal ratio (Log 16S copies/ITS copies) in the two mixtures (Mix1 and Mix2) of forest litters (WO: white oak, OP: holm oak and Aleppo pine, BB: bamboo, CN: chestnut, HB: hackberry, HO: holm oak, MP: maritime pine, Mix: mixture of litters before fermentation, FMix: mixture of litters after fermentation). Different letters above the boxes indicate significant differences (*p* < 0.001) among litters. Figure S3. Relationship between the bacterial to fungal ratio (16S copies/ITS copies) and the C to N ratio of the different forest litters. A significant non-linear regression was found (*p* < 0.001, r^2^ = 0.74). Figure S4. Differences in bacterial TRF of fungal ITS amplicon number in the two mixtures (Mix1 and Mix2) of forest litters (WO: white oak, OP: holm oak and Aleppo pine, BB: bamboo, CN: chestnut, HB: hackberry, HO: holm oak, MP: maritime pine, Mix: mixture of litters before fermentation, FMix: mixture of litters after fermentation). Different letters above the boxes indicate significant differences (*p* < 0.001) among litters. Figure S5. Differences in bacterial, fungal and chemical composition of the two mixtures before (Mix1 and Mix2) and before (FMix1 and FMix2) after fermentation. Between Class Analysis (BCA) is based on bacterial (above) or fungal (middle) molecular fingerprints or MIR spectra (below). The ellipses represent 60% of the variability of a litter. Letters represent the barycenter of the replicates (n = 3) for each treatment. Monte Carlo test simulated *p* values (lower right corner) revealed significant differences among litters.
