# Supplementary material for: Chemical and Microbial Characterization of Fermented Forest Litters Used as Biofertilizers
Source: Microorganisms. 2023 Jan 24;11(2):306. doi: 10.3390/microorganisms11020306 (PMC9959058; doi:10.3390/microorganisms11020306)
Supplement: Supplementary file 1 [file microorganisms-11-00306-s001.zip › microorganisms-2045812-supplementary.pdf]

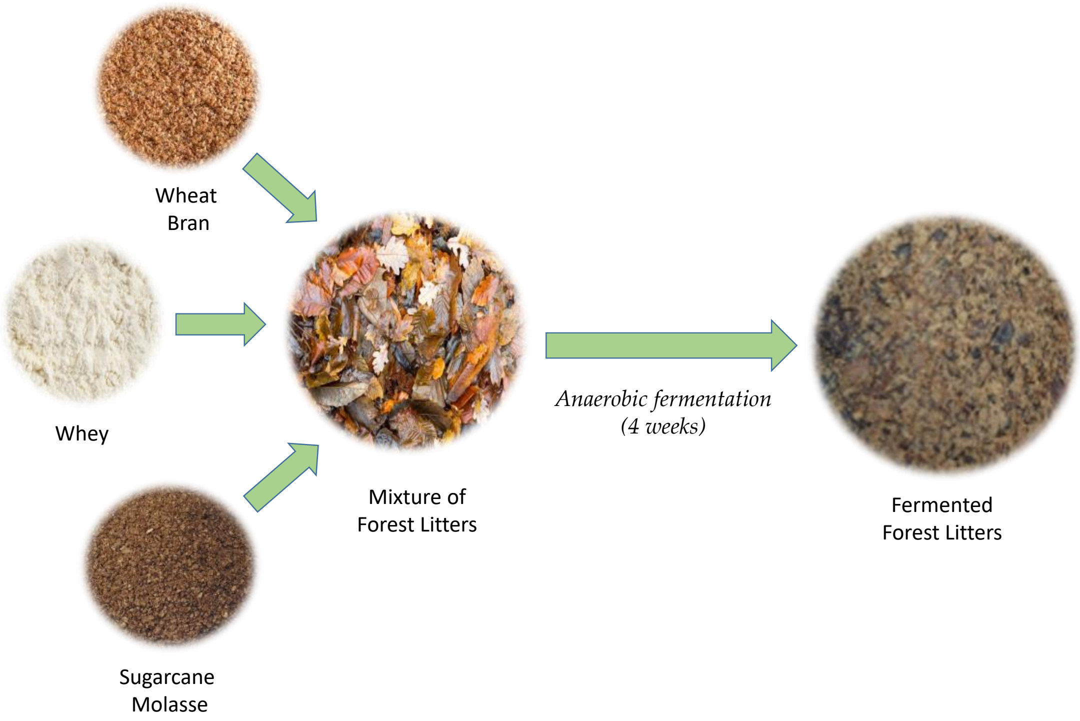

Figure S1. Flowchart representing the different steps of the process, from the mixture of forest litters, addition of wheat bran, whey and sugarcane molasses, to the anaerobic fermentation during 4 weeks.

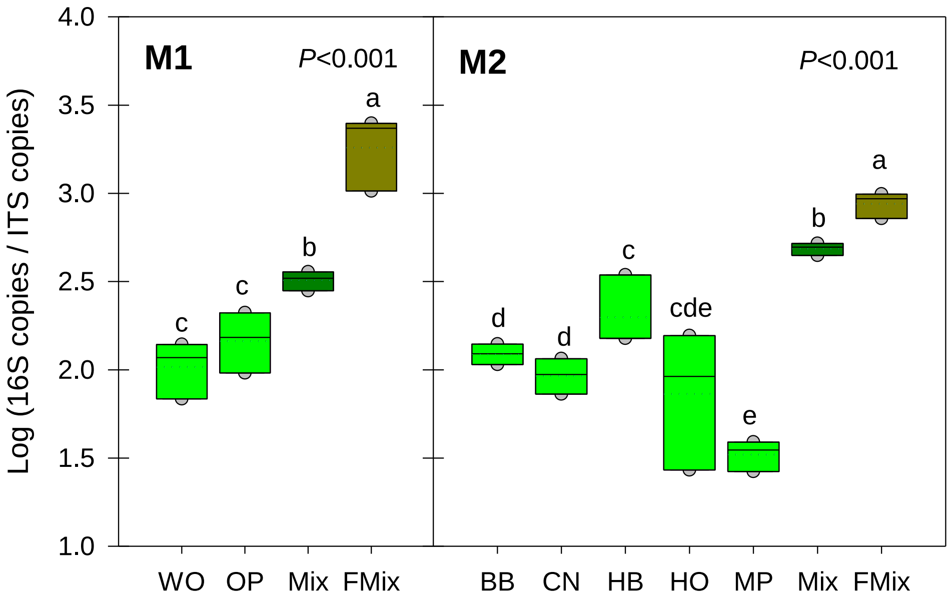

Figure S2. Differences in bacterial to fungal ratio (Log 16S copies / ITS copies) in the two mixtures (Mix1 and Mix2) of forest litters (WO: white oak, OP: holm oak and Aleppo pine, BB: bamboo, CN: chestnut, HB: hackberry, HO: holm oak, MP: maritime pine, Mix: mixture of litters before fermentation, FMix: mixture of litters after fermentation). Different letters above the boxes indicate significant differences ($P<0.001$) among litters.

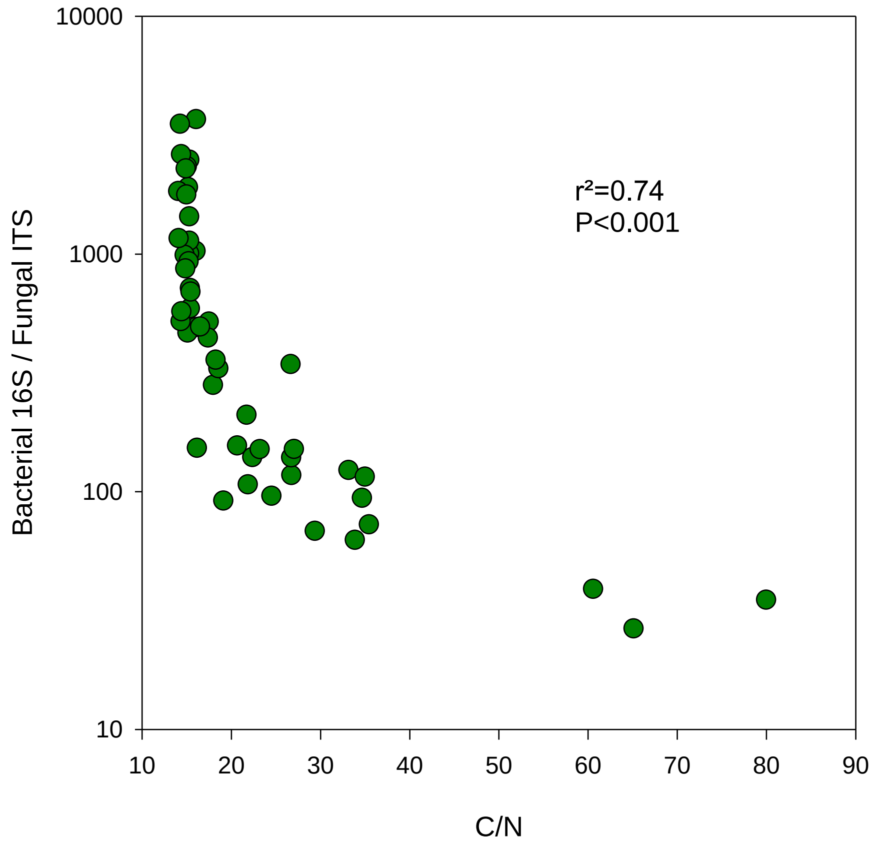

Figure S3. Relationship between the bacterial to fungal ratio (16S copies /ITS copies) and the C to N ratio of the different forest litters. A significant non-linear regression was found ($P<0.001$, $r^2=0.74$).

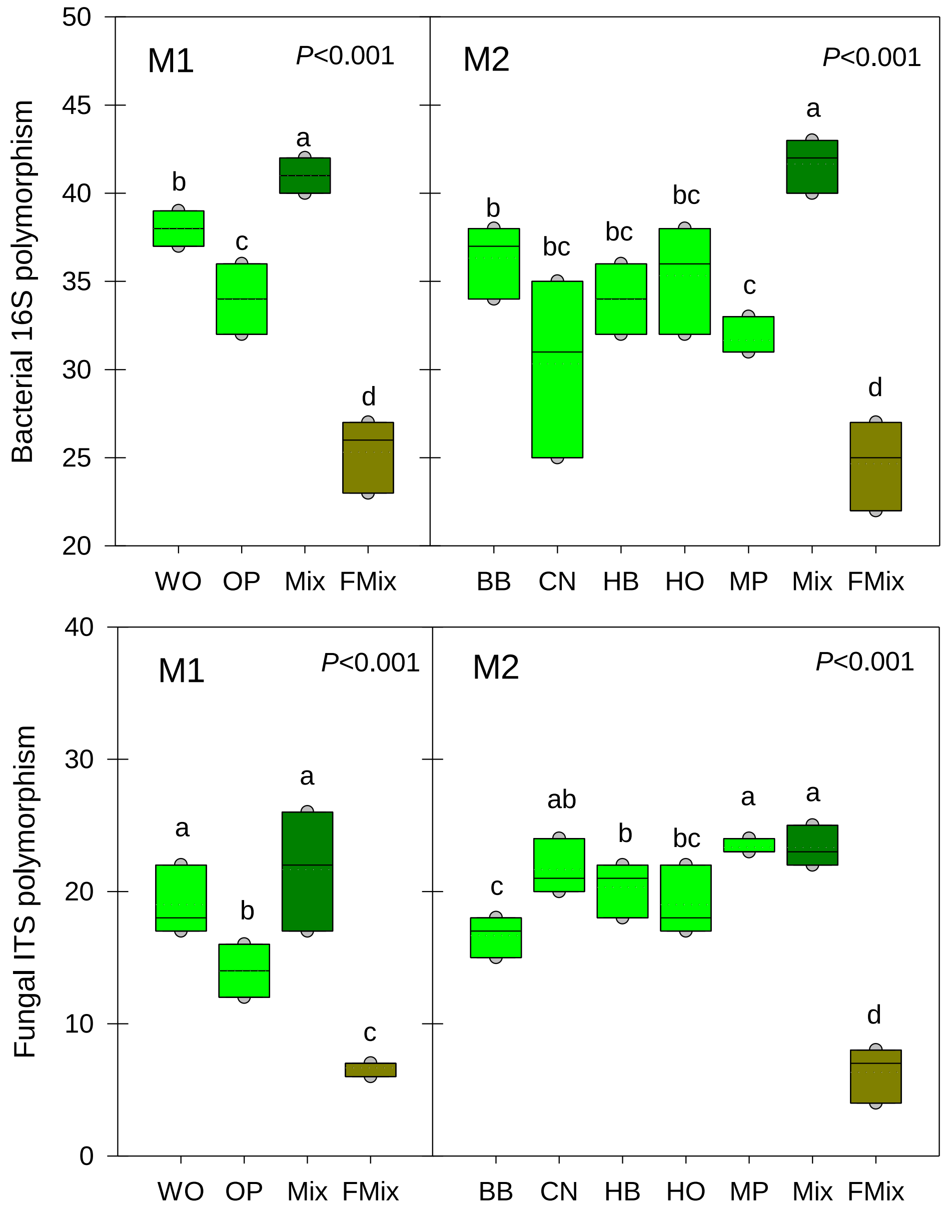

Figure S4. Differences in bacterial TRF of fungal ITS amplicon number in the two mixtures (Mix1 and Mix2) of forest litters (WO: white oak, OP: holm oak and Aleppo pine, BB: bamboo, CN: chestnut, HB: hackberry, HO: holm oak, MP: maritime pine, Mix: mixture of litters before fermentation, FMix: mixture of litters after fermentation). Different letters above the boxes indicate significant differences (P<0.001) among litters.

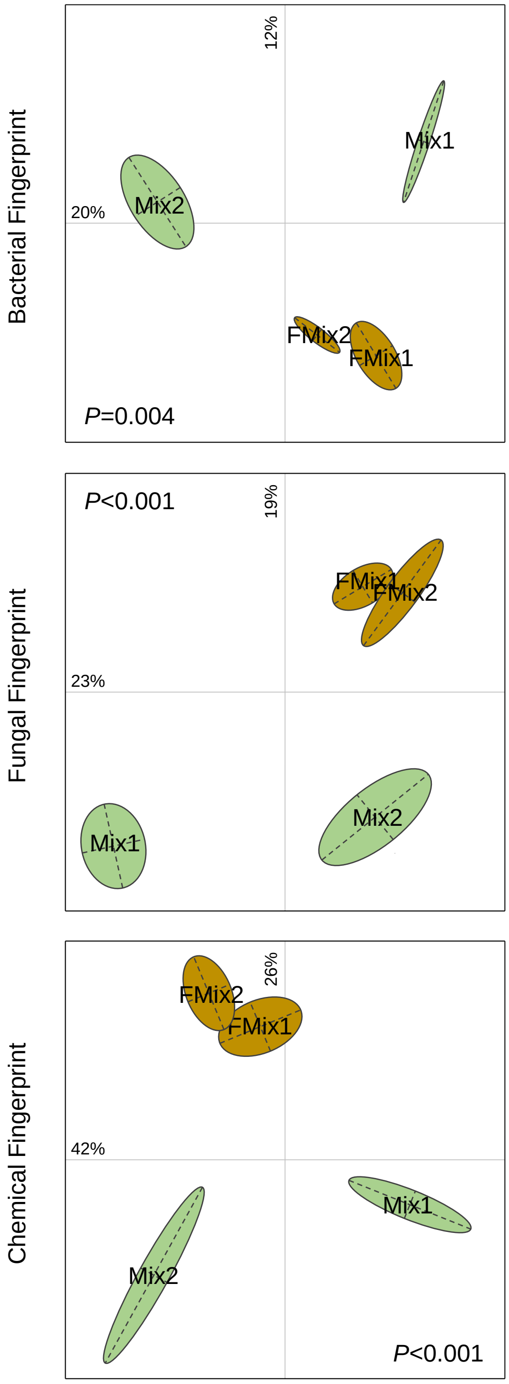

Figure S5. Differences in bacterial, fungal and chemical composition of the two mixtures before (Mix1 and Mix2) and before (FMix1 and FMix2) after fermentation. Between Class Analysis (BCA) are based on bacterial (above) or fungal (middle) molecular fingerprints or MIR spectra (below). The ellipses represent 60% of the variability of a litter. Letters represent the barycenter of the replicates (n=3) for each treatment. Monte Carlo test simulated P values (lower right corner) revealed significant differences among litters.
